# Mesoporous Silica Nanoparticles Mediate SiRNA Delivery for Long‐Term Multi‐Gene Silencing in Intact Plants

**DOI:** 10.1002/advs.202301358

**Published:** 2023-12-25

**Authors:** Yao Cai, Zhujiang Liu, Hang Wang, Huan Meng, Yuhong Cao

**Affiliations:** ^1^ Key Laboratory for Biomedical Effects of Nanomaterials and Nanosafety National Center for Nanoscience and Technology Chinese Academy of Sciences Beijing 100190 China; ^2^ School of Nano Science and Technology University of Chinese Academy of Sciences Beijing 100049 China

**Keywords:** gene silencing, mesoporous silica nanoparticles, plant biotechnology, siRNA delivery

## Abstract

RNA interference (RNAi) is a powerful tool for understanding and manipulating signaling pathways in plant science, potentially facilitating the accelerated development of novel plant traits and crop yield improvement. The common strategy for delivering siRNA into intact plants using agrobacterium or viruses is complicated and time‐consuming, limiting the application of RNAi in plant research. Here, a novel delivery method based on mesoporous silica nanoparticles (MSNs) is reported, which allows for the efficient delivery of siRNA into mature plant leaves via topical application without the aid of mechanical forces, achieving transient gene knockdown with up to 98% silencing efficiency at the molecular level. In addition, this method is nontoxic to plant leaves, enabling the repeated delivery of siRNA for long‐term silencing. White spots and yellowing phenotypes are observed after spraying the MSN‐siRNA complex targeted at phytoene desaturase and magnesium chelatase genes. After high light treatment, photobleaching phenotypes are also observed by spraying MSNs‐siRNA targeted at genes into the Photosystem II repair cycle. Furthermore, the study demonstrated that MSNs can simultaneously silence multiple genes. The results suggest that MSN‐mediated siRNA delivery is an effective tool for long‐term multi‐gene silencing, with great potential for application in plant functional genomic analyses and crop improvement.

## Graphical abstract

A biocompatible mesoporous silica nanoparticles (MSNs) platform for siRNA delivery into mature plant cells is established, which allows long‐term and multi‐gene silencing of both exogenous and endogenous genes through foliar spraying of MSN‐siRNA complexes, resulting in obvious phenotypic changes in the plant. This technology has great potential for applications in plant functional genomic analyses and crop improvement.

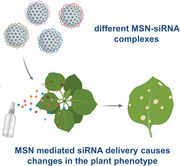

## 1 Introduction

Advances in genomic sequencing have made complete plant genome sequencing possible, bringing a new dimension to genetic modification for developing novel plant traits and improving crop yield.^[^ ^1^ ^]^ A major challenge in applying this large plant genomic information base for plant improvement is the determination of gene function in the plant genome and mapping out the links from genome type to phenotype.^[^ ^2^ ^]^ Transgenic technologies permit the introduction of genes of interest into plants, providing a powerful tool for understanding their genetic functions. However, these technologies often require prior identification of specific genes. Furthermore, traditional methods for gene identification include plant transformations involving Agrobacterium or a gene gun for DNA plasmid delivery. These processes are labor‐intensive, time‐consuming, and species‐specific,^[^ ^3^ ^]^ limiting their potential for large‐scale genetic screening and multi‐gene targeting. Although the development of RNA interference (RNAi) technology offers a new avenue for studying gene function by direct gene silencing,^[^ ^4^ ^]^ it faces several intracellular delivery challenges that hinder its further application.^[^ ^5^, ^6^ ^]^

Nanomaterials have several unique advantages for delivering genetic materials into mature plants. Owing to their extremely small size and unique physical and chemical properties, they can pass through cell walls and release their contents inside cells.^[^ ^7^ ^]^ Recent studies have shown that carbon nanotubes can deliver GFP‐expressing DNA plasmids and siRNAs to mature plant leaves via infiltration. This demonstrates direct DNA transformation and gene silencing of intact plant cells.^[^ ^5^, ^8^, ^9^ ^]^ Mitter et al. revealed that clay nanosheets could facilitate the delivery of pathogen‐specific double‐stranded RNA into intact plant cells via topical application to overcome viral resistance on plant surfaces.^[^ ^10^ ^]^ Other nanostructures, including graphene oxide nanoparticles,^[^ ^11^ ^]^ gold nanoclusters,^[^ ^3^ ^]^ carbon dots,^[^ ^12^ ^]^ and DNA origami^[^ ^13^ ^]^ have also been used for siRNA delivery into intact plants. This progress has further advanced the application of RNAi in plant science research.

Silica‐based materials have been approved to be used as food additives by the U.S. Food and Drug Administration.^[^ ^14^ ^]^ Mesoporous silica nanoparticles (MSNs) are silicon dioxide nanoparticles that have mesopores, they are biocompatible and have been widely used in animal research to deliver small‐molecule drugs and genetic materials.^[^ ^15^ ^]^ MSNs feature several advantages, making them good candidates for a wide range of bioengineering applications. For example, the sol‐gel method of MSN preparation in an aqueous solution is inexpensive, simple, and easy to scale up. In addition, MSNs can be easily functionalized with various chemical groups.^[^ ^16^ ^]^

Recent studies have shown that MSNs can facilitate GFP‐encoded DNA plasmid transformation into intact *Arabidopsis thaliana* roots.^[^ ^17^ ^]^ Gold‐functionalized MSNs mediated the co‐delivery of DNA plasmids and proteins into intact plant cells via biolistic bombardment.^[^ ^18^, ^19^, ^20^ ^]^ With ultrasound assistance, Wang et al. showed that poly L‐lysine‐functionalized MSNs could facilitate DNA transformation in tobacco leaves.^[^ ^21^ ^]^ Other groups have reported that ultrafine MSNs in the 5–20 nm range can deliver small molecules, such as fluorescein isothiocyanate^[^ ^22^ ^]^ and salicylic acid^[^ ^23^ ^]^ into intact plant cells. These findings suggest that MSNs may be ideal candidates for mediating siRNA delivery and gene silencing in intact plants.

**Figure 1:**
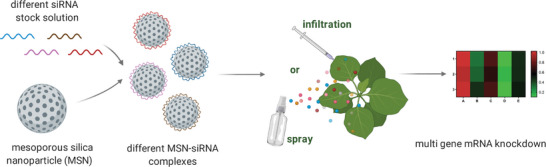
Schematic representation of MSN‐mediated siRNA delivery for multi‐gene silencing in intact plants.

Here, we demonstrated for the first time that via topical application without the aid of mechanical forces, MSN‐mediated siRNA delivery can effectively silence targeted genes in mature plant leaves with up to 98% silencing efficiency (**Figure** **1**). Unlike infiltration techniques that require careful leaf‐by‐leaf injection, foliar spraying provides a simple and user‐friendly application method, thus enabling sequential and multi‐targeting MSNs‐siRNA silencing. We knocked down six endogenous target genes. The silencing effects were exhibited phenotypically and at the molecular level. In particular, we observed a sequential silencing of the targeted genes, that silence multiple genes simultaneously. These results demonstrate that MSN‐mediated siRNA delivery can be an effective tool for long‐term multi‐gene silencing and can be applied in plant functional genomic analyses and crop improvement.

## 2 Results and Discussion

### 2.1 Analysis of the Association of the MSN‐siRNA Complex with Plant Leaf Cells

**Figure 2:**
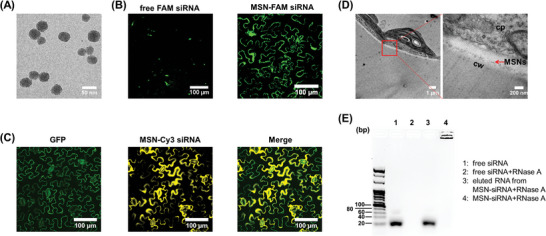
The interaction of MSNs with *N. benthamiana* leaves. A) TEM images of synthesized MSN selected in this study (MSN 01, 31 nm); B) Representative confocal microscopy images of *N. benthamiana* leaves infiltrated with MSN 01 loaded with FAM‐modified scrambled siRNA and free FAM‐modified scrambled siRNA. The fluorescence was collected to analyze the distribution of MSNs in leaves. C) Confocal microscopy imaging in a GFP transgenic 16 C line; *N. benthamiana* plant infiltrated with MSN‐loaded Cy3‐siRNA. D) Representative TEM images of *N. benthamiana* leaves 24 h postinfiltration with siRNA‐functionalized MSN 01. Scale bars: 1 µm. The images show magnifications on the right, with the red boxes indicating the areas of magnification. Scale bars: 200 nm. The red arrows indicate MSNs. Annotations represent cell wall (cw) and cytoplasm (cp). (E) siRNA was protected by MSN. The lanes from 1 to 4 stand for free siRNA, free siRNA with RNase A treatment, siRNA desorbed from MSN‐siRNA after RNase A treatment for 1 h at 37 °C, and MSN incubated with siRNA, then treated with RNase A.

To explore the size‐dependent ability of MSNs transit within plant tissues, we synthesized four types of MSNs of different sizes using a positively charged cetyltrimethylammonium bromide template and NH_4_OH at different concentrations (**Figure** **2**A; Figure S1 and S2A–C, Supporting Information) and examined the association between these MSNs and plant cells using fluorescence microscopy analysis to observe the distribution of the dye‐labeled siRNA‐MSN complex (MSNs‐siRNA) in mature plants. The optimal siRNA‐loading capacity of MSNs of different sizes was estimated using gel electrophoresis (Figure S3, Supporting Information). Notably, MSNs without any chemical modification could not bind to siRNA (Figure S3E, Supporting Information), suggesting that the siRNA loading mechanism is via static force as APTS‐modified MSN is positively charged (Figure S3F, Supporting Information). To investigate the effect of siRNA‐loaded MSNs on intact plant leaf cells, we infiltrated FAM‐labeled siRNA‐MSNs at the optimal siRNA: MSN mass ratio into *Nicotiana benthamiana* leaves.

Twenty‐four hours after infiltration, a small area of the infiltrated leaf was cut for confocal microscopy. The fluorescence signal from the 31 nm FAM‐labeled siRNA‐MSNs was clearly observed along the cell contour, whereas the same signal was not observed in the free FAM‐labeled siRNA group (Figure 2B). The fact that the signal was not observed in the other three large‐sized MSNs loaded with siRNA along the cell contour (Figures S2D–F, Supporting Information), indicates that MSNs with an average diameter of 31 nm have more potential in delivering siRNA into leaf cells. To further test the interaction between MSN‐siRNAs and intact *N. benthamiana* leaf cells, we studied 16C GFP transgenic *N. benthamiana* using Cy3‐labeled siRNA‐MSNs. Following 24 h of incubation leaves treated with Cy3‐siRNA‐MSNs showed a high degree of colocalization between the intracellular (cytosolic) GFP and Cy3 fluorescence originating from the siRNA‐MSNs complex (Figure 2C). The percentage of the colocalization between the Cy3 signal and GFP signal analyzed by Image J was 25%, confirming the ability of MSN‐siRNAs to diffuse within the leaf interstitial space and to associate with plant leaf cells.

We also applied transmission electron microscope (TEM) analysis to directly visualize the MSNs distribution in the leaf of *N. benthamiana* (Experimental Section). We did not clearly observe siRNA‐loaded 31 nm MSNs in the intracellular space of *N. benthamiana* leaves. This was possibly due to the difficulty in distinguishing small‐sized MSNs from ribosomes owing to their low electron density in ultrathin TEM sections of the plant material. However, siRNA‐loaded 31 nm MSNs formed a strong interaction with the cell wall and were evenly distributed along the cell wall (Figure 2D), compared to the untreated leaves that showed clear and smooth cell wall boundaries (Figure S4, Supporting Information). Larger‐sized MSNs were easier to identify under TEM. We observed that these MSNs were blocked at the cell wall (Figure S2G–I, Supporting Information). Studies have shown that the size exclusion principle of plant cell walls does not always apply to the successful delivery of siRNA^[^ ^24^, ^25^, ^26^, ^27^ ^]^; however, the interaction between the nanoparticles and the cell wall observed using TEM seems to be a good indicator of successful gene silencing.^[^ ^11^, ^25^ ^]^ In particular, nanoparticles must intercalate into the plant cell wall for efficient siRNA silencing.^[^ ^25^ ^]^ Based on these results, we selected 31 nm MSNs for siRNA delivery.

The MSN‐siRNA complex can facilitate siRNA internalization and protect siRNA from RNase A degradation. We performed gel electrophoresis to determine the siRNA degradation level after RNase A treatment (Experimental Section). Compared to untreated siRNA, siRNA incubated with RNase A did not show an observable band, suggesting the degradation of free RNA by RNase A (Figure 2E, lanes 1 and 2). Once siRNA was loaded onto MSN and treated with RNase A, the MSN‐bound siRNA did not migrate from the well during electrophoresis and presented as fluorescence in the well (Figure 2E, lane 4). When MSN‐siRNA was dissociated at 95 °C after the RNase A treatment, a positive band was observed during electrophoresis, indicating that MSNs can protect RNA from enzymolysis (Figure 2E, lane 3). A possible reason for such MSN protection is that the dense shell of oligonucleotides on the surface of nanoparticles inhibits their degradation by nucleases.^[^ ^28^ ^]^

MSNs are biocompatible, hydrolytically unstable, and dissolvable over time into water‐soluble silicic acid.^[^ ^29^ ^]^ Research has shown that amine group‐modified MSNs can be degraded in both neutral and acidic ambient solutions.^[^ ^30^ ^]^ We incubated MSNs in *N. benthamiana* leaf cell lysate solution at room temperature to determine the degradation rate (Experimental Section). The samples were observed using TEM at the designated time points. The synthesized MSNs appeared as intact spheres with clear boundaries and a pore architecture before exposure to the leaf cell lysate solution. However, they appeared eroded with the loss of the initial spherical shape after 1 day of cell lysate exposure; the particles remained degraded and became irregularly fused after 8 days of treatment (Figure S5, Supporting Information).

### 2.2 MSN‐siRNA Gene Silencing in Plant Cells By Foliar Spraying

We attempted gene silencing of MSN‐siRNA by infiltrating GFP‐transgenic *N. benthamiana*, as the GFP gene is a widely used reporter in transient assays for evaluating gene silencing effects. The expression level of *GFP* mRNA was quantified 1 day after infiltration using quantitative polymerase chain reaction (qPCR). Reverse transcription‐qPCR results demonstrated a 62% reduction (Figure S6A, Supporting Information) in *GFP* mRNA expression levels in the MSN‐siRNA‐treated leaves. Foliar spraying was also used to apply the MSN‐siRNA solution to the leaves. TEM analysis revealed that MSNs were mainly attached to the exterior of the cell wall, suggesting the affinity of the MSN‐siRNA complex for the plant cell wall, compared to untreated leaves (Figure S6B, Supporting Information). One day after spraying the MSN‐siRNA complex, *GFP* expression was reduced by 66% (Figure S6C, Supporting Information), comparable to the silencing via infiltration. We also evaluated the gene‐silencing efficiency of MSN‐siRNA by direct silencing of *ROQ1*, an endogenous plant gene that resists certain plant pathogens. The *ROQ1* mRNA transcription levels of the MSN‐siRNA‐treated leaves via infiltration or spraying showed 46% or 56% (Figure S7, Supporting Information) reduction in the *ROQ1* mRNA transcription levels, respectively. The loading buffer solution, free siRNA, or MSNs alone did not influence the *ROQ1* mRNA levels in *N. benthamiana* leaves with either application method because both methods provided a similar MSN‐siRNA delivery outcome. However, the foliar spraying method is more user‐friendly and time‐saving. Thus, we used foliar spraying for all the subsequent silencing experiments.

**Figure 3:**
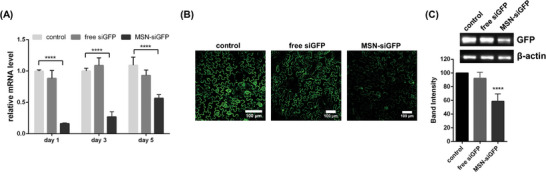
MSNs mediated *GFP* gene silencing by spraying. A) qPCR analysis for GFP mRNA fold changes at 1, 3, and 5 days post‐treatment by spraying MSN‐siGFP. B) Representative confocal microscopy images of control, free siGFP, and MSN‐siGFP treated 16 C line leaves. All scale bars, 100 µm. C) Representative western blot for GFP extracted from control, free siGFP, and MSN‐siGFP delivered 16 C line leaves at 3 days post‐treatment (The plant β‐actin as the reference protein). ^***^ *p* < 0.001 and ^****^ *p* < 0.0001 are significant using a one‐way ANOVA test. Data represent mean ± sem.

MSN‐mediated GFP‐targeting siRNA was sprayed onto GFP transgenic *N. benthamiana* to further confirm the gene expression at the protein level since confocal microscopy analysis can be used to observe the fluorescence directly, and western blotting can be used to analyze the protein expression. The GFP mRNA transcription levels were analyzed at 1, 3, and 5 days after treatment, and we observed that the GFP gene was significantly knocked down 1 day after treatment until 5 days post‐treatment (**Figure** **3**A). The MSN‐siGFP‐treated leaves exhibited significantly reduced expression at 3 days post‐treatment compared to the control and free siGFP‐treated leaves, as revealed by confocal microscopy images (Figure 3B).

In addition, we analyzed the GFP protein expression level in intact leaves at 1, 3, and 5 days post‐treatment using western blotting, where GFP from MSN‐siGFP‐treated leaves was significantly lower than that from the control and free siGFP‐treated leaves at 3 days post‐treatment (Figure S8A, Supporting Information). Band intensity analysis revealed a 36% reduction of the GFP expression in MSN‐siGFP‐treated leaves compared to the control (Figure 3C). Plant β‐actin was used as the reference protein and maintained the same expression across treatments during the experiment (Figure S8B, Supporting Information).

### 2.3 MSN‐Mediated Endogenous Gene Silencing and Phenotypical Changes in Mature Plant Leaves

To confirm that MSN‐mediated siRNA silencing is universally applicable for silencing other genes, we first tested whether our platform could efficiently silence two frequently studied genes, *PDS* and *ChlH*. *PDS* is an important rate‐limiting enzyme in carotenoid synthesis that converts phytoene to colored ξ‐carotene during carotenoid metabolism and photosynthesis. The albinism caused by *PDS* silencing, which is evidently observed in plant leaves, indicates a change in the concentration of photosynthetic pigments. *ChlH* encodes the H subunit of the magnesium chelatase required for chlorophyll production. *ChlH* silencing in plants results in a yellowing phenotype due to the disruption of chlorophyll biosynthesis.

**Figure 4:**
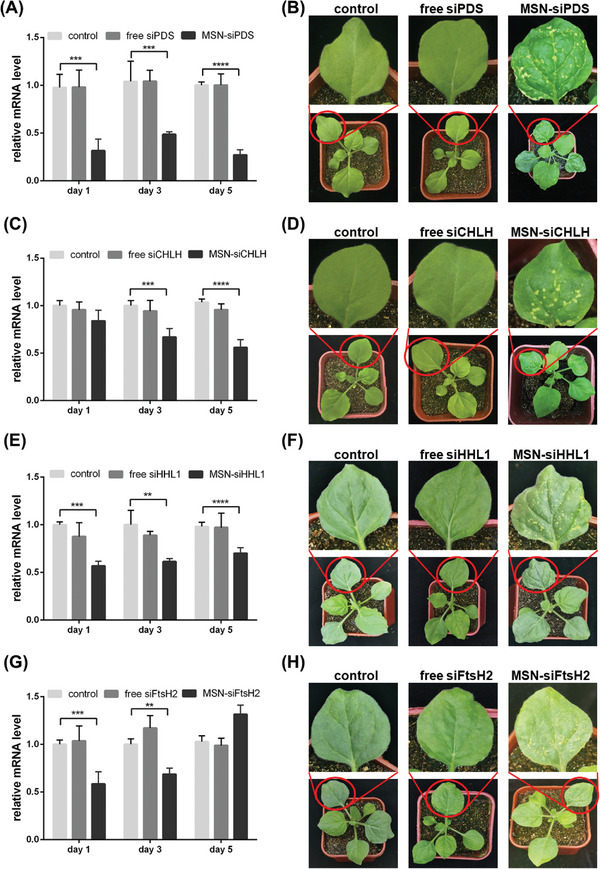
MSNs mediated endogenous gene silencing by spraying. siRNA delivered to leaves by MSNs via spraying, the A) PDS, C) ChlH, E) HHL1, and G) FtsH2 genes were silenced effectively compared with the relative controls as measured by qPCR analysis. (B), (D), (F), and (H) show the corresponding phenotype changes. ^**^ *p* < 0.01,^***^ *p* < 0.001 and ^****^ *p* < 0.0001 are significant using one‐way ANOVA test. Data represent mean ± sem.

We harvested *N. benthamiana* leaves 1, 3, and 5 days after spraying with MSN‐siRNA. The *PDS* gene was significantly knocked down 1 day after treatment until 5 days post‐treatment (**Figure** **4**A), whereas the *ChlH* gene was significantly knocked down 3 days post‐treatment until 5 days post‐treatment (Figure 4C). The most visible albinism phenotype caused by MSN‐mediated *PDS* silencing was observable on tobacco leaves at 11 days postspraying (Figure 4B), indicating a change in the concentration of photosynthetic pigments. *ChlH* silencing in the plant resulted in the most visible yellow spot phenotype at 9 days postspraying due to the disruption of chlorophyll biosynthesis (Figure 4D).

To further demonstrate the silencing versatility of the MSN platform, we selected two other endogenous genes, *HHL1* and *FtsH2*, to assess whether silencing these genes could lead to phenotypical changes under extreme environmental conditions. Both *HHL1* and *FtsH2* play important roles in PSII protection and repair.^[^ ^31^ ^]^ Their silencing can cause photodamage in plants under high light exposure, thereby inducing leaf blade bleaching.

We harvested *N. benthamiana* leaves 1, 3, and 5 days after spraying with MSN‐siRNA. Both genes were significantly knocked down 1 day after MSN‐siRNA treatment (Figure 4E,G). The transcription level of *HHL1* was suppressed for more than 5 days postspraying, whereas that of *FtsH2* was suppressed until day 3 and returned to normal levels on day 5 post‐treatment, suggesting different silencing responses from different genes. Moreover, since both genes play key roles in maintaining the dynamic equilibrium of the PSII system,^[^ ^32^ ^]^ silencing either can lead to photobleaching in the phenotypic plant leaves. After MSN‐siRNA spraying and high light treatment (Experimental Section), both MSN‐siHHL1 and MSN‐siFtsH2 treated plant leaves exhibited a visible photobleaching phenotype from day 5 (Figure 4F,H). The photobleaching phenotype manifested as yellowish spots on the leaves. Notably, the highlight treatment lasted for 14 days. The photobleaching phenotype by day 14 did not recover from its state on day 5, suggesting that photodamage might occur at a specific stage in the PSII repair cycle while maintaining dynamic equilibrium in the plant. The MSN‐siRNA‐treated leaves showed no observable changes without high light exposure (Figure S9, Supporting Information), consistent with the well‐understood characteristics of *HHL1* and *FtsH2*.

The control and free siRNA groups (Figure 4) indicated that siRNA was the key factor of the photobleaching phenotype, as the other chemicals did not cause phenotypic changes.

### 2.4 Sequential Delivery of siRNA for Long‐Term Silencing

Differentially expressed genes, such as those expressed during cotton fiber development, require continuous knockdown of hub genes in research applications to reveal genetic and molecular mechanisms.^[^ ^33^ ^]^ We studied whether sequential MSN‐siRNA spray treatments could lead to long‐term silencing. Before this study, siRNAs of *SOS* were also incubated with MSN and sprayed on *N. benthamiana* leaf. The *SOS* gene is involved in the signaling pathway in response to salt stress, an example of abiotic stress. The high light treatment served as a test of abiotic stress to examine whether *SOS* silencing could reduce abiotic stress resistance in treated plants. *SOS* was significantly knocked down 1 day after *N. benthamiana* leaves were sprayed with MSN‐siSOS until 3 days postspray application (Figure S10, Supporting information). Subsequently, *SOS, FtsH2, HHL1*, and *GFP* coupled with MSNs were sprayed every other day for 14 days (days 0–13) to analyze the long‐term silencing effect.

**Figure 5:**
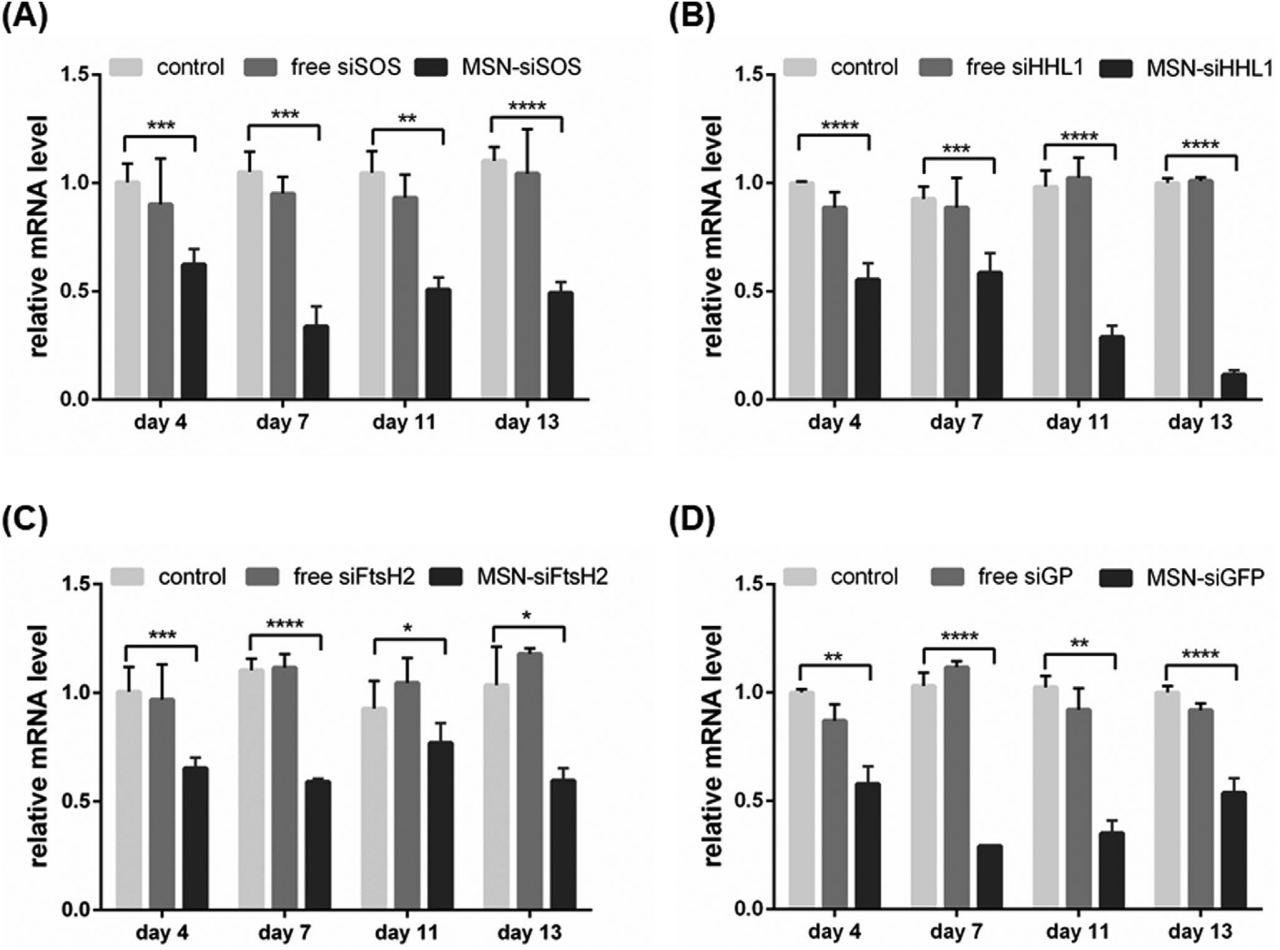
MSNs mediated effective long‐term gene silencing. *N. benthamiana* leaves were sequentially sprayed with MSN‐siRNA thrice weekly for 2 weeks. The leaves were collected on days 4, 7, 11, and 13. qPCR analysis of fold changes for four mRNAs A) SOS, B) HHL1, C) FtsH2, and D) GFP. ^*^ *p* < 0.05, ^***^ *p* < 0.001 and ^****^ *p* < 0.0001 are significant using one‐way ANOVA test. Data represent mean ± sem.

After spraying, the leaves were harvested and analyzed on days 4, 7, 11, and 13. All four targeted genes were knocked down at each sampling time point, and each gene showed consistently low expression levels over the experimental period (**Figure** **5**A–D). Notably, all four genes with different physiological functions showed ≈50% mRNA expression efficiency on day 4. However, the expression level of the four genes subsequently progressed differently. The three subsequent sampling time points revealed stable and evident silencing effects for *SOS* and *GFP* expression. The expression level of *HHL1* continuously decreased to ≈12.5% mRNA expression efficiency by the end of the silencing period. Interestingly, *FtsH2* showed ≈50% mRNA expression efficiency at the first two sampling time points and slight expression recovery at subsequent sampling times. *FtsH2* has been used in a physiological role in studies on D1 degradation of PSII repair^[^ ^34^ ^]^ and damaged D1 removal by UV–B irradiation and heat^[^ ^35^, ^36^ ^]^ and plays a role in osmoprotection.^[^ ^37^ ^]^ The multiple functions of *FtsH2* in vivo may be responsible for its higher resistance to silencing compared to the other candidate genes.

### 2.5 Multiple Genes Knocked Down by MSN‐siRNA Spray Treatment

**Figure 6:**
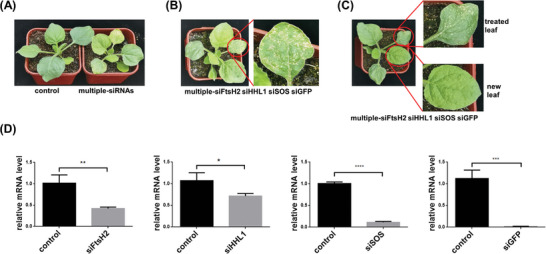
MSNs mediated effective multi‐gene silencing. A) The phenotype of *N. benthamiana* seedlings treated with control (scrambled siRNA loaded MSN) or MSNs‐siRNA that targeted multiple genes. B) The piebald phenotype of *N. benthamiana* leaves with multiple MSN‐siRNAs (siFtsH2, siHHL1, siSOS, siGFP) silencing. C) The phenotype of leaves with multiple MSN‐siRNAs silencing and new leaves. D) qPCR analysis of fold changes for four mRNAs (FtsH2, HHL1, SOS, and GFP) after spraying the multiple MSN‐siRNAs. ^*^ *p* < 0.05, ^**^ *p* < 0.01, ^***^ *p* < 0.001 and ^****^ *p* < 0.0001 are significant using one‐way ANOVA test. Data represent mean ± sem.

Multiple genes or gene homologs control many agronomically important traits, particularly in multiploid plants.^[^ ^38^ ^]^ To estimate whether MSNs could simultaneously deliver different gene‐targeting siRNAs into leaves, we sprayed *N. benthamiana* leaves every 2 days for 6 days with a mixture of four different MSN‐siRNAs targeting *FtsH2, HHL1, SOS*, and *GFP*. The seedlings treated with multiple target genes of MSNs‐siRNA were shorter and smaller and showed etiolation after the highlight treatment (**Figure** **6**A). The multiple MSN‐siRNA‐treated mature leaves also exhibited observable phenotypic changes. Notably, in comparison with single‐gene silencing, multiple MSN‐siRNA‐treated plants presented a more heavily photobleaching phenotype (Figure 6B). *HHL1* and *FtsH2* silencing can induce leaf blade bleaching. *SOS* silencing can reduce abiotic stress resistance reflected by shorter and etiolated seedlings in treated plants and enhance the *HHL1* and *FtsH2* silencing effects under high‐light treatment. The stronger photobleaching phenotype could result from the stacking effect induced by *SOS, FtsH2*, and *HHL1* silencing, limiting plant growth after photoinhibition. Moreover, the newly grown leaves unsprayed with MSN‐siRNAs were healthy and showed no phenotypic differences compared to the control (Figure 6C). The leaves treated with MSNs alone or the buffer solution showed no phenotypic changes (Figure S11, Supporting Information).

We further analyzed the targeted gene expression of the multiple MSN‐siRNA‐treated leaves at the molecular level. Leaves were harvested on day 7. The mRNA expressions of the four individual genes were detected using digital PCR and qPCR, and all four genes were efficiently knocked down. The expressions of *GFP, SOS, FtsH2*, and *HHL1* in MSN‐siRNA treated leaves were 9.46, 3.16, 2514.22, and 2365.21 copies ul^−1^, respectively. Their expressions in the control group were 58.96, 58.20, 4992.08, and 5207.22 copies ul^−1^, respectively (Figure S12, Supporting Information). The knockdown efficiency of each gene measured by qPCR was consistent with the results obtained from digital PCR, in which GFP was knocked down by ≈98%, *SOS* by ≈90%, and *FtsH2* and *HHL1* by 40–50% (Figure 6D). In our study, *GFP* and *SOS* were the two genes that were relatively easily knocked down, which is possibly due to the high specificity of the selected siRNA sequence. When the multi‐gene experiment was conducted by spraying the MSN‐siRNA complex mixture every 2 days, the gene expression levels were continuously suppressed, indicating extremely low silencing efficiency.

## 3 Conclusion

We have established a biocompatible MSNs platform for siRNA delivery into mature plant cells. The delivery platform allows the effective silencing of both exogenous and endogenous genes, with up to 98% silencing efficiency. MSN‐siPDS‐ and MSN‐siChlH‐treated *N. benthamiana* leaves showed evident white spots and yellowing phenotypic changes, respectively. MSN‐siFtsH2 or MSN‐siHHL1 treated plant leaves exhibited a photobleached phenotype after exposure to high light intensity. Using the foliar spray method, we demonstrated that MSNs enable sequential and multitargeted siRNA delivery. Repeated MSN delivery of different siRNAs for 14 days led to the continuous knockdown of the targeted genes. Interestingly, the multi‐gene targeting MSN‐siRNA‐treated plants presented as shorter plants with a more intensely photobleached phenotype, which we hypothesized resulted from a stacking effect induced by the simultaneous silencing of *SOS, FtsH2*, and *HHL1*. Given its versatility, we believe the MSN‐siRNA delivery platform has a broad range of applications in various studies. For instance, to identify gene function and regulation during the developmental period of a specific plant, or RNAi‐based plant disease and insect resistance. Thus, the MSN‐siRNA delivery platform can be a powerful tool in plant biotechnology.

## 4 Experimental Section

### Materials

Cetyltrimethylammonium bromide (CTAB, ≥99%), tetraethyl orthosilicate (TEOS, 98%), 3‐aminopropyltriethoxysilane (APTS, ≥98%) were purchased from Sigma‐Aldrich. Hydrogen chloride (HCl, 36.5–38%, trace metal grade) was purchased from China National Pharmaceutical Group Corporation, sodium hydroxide (NaOH, 97%) was purchased from Sangon Biotech (Shanghai) Co., Ltd., Ethanol and magnesium chloride hexahydrate was purchased from Beijing Innochem Science & Technology co., LTD., Nuclease‐free water was purchased from Beyotime Biotechnology, MES hydrate was purchased from Amresco, cDNA synthesis kit and SYBR Green Master Mix were purchased from Nanjing Vazyme Biotech Co., Ltd., RNase A was purchased from Omega Bio‐Tek, The antibodies used for these immunological experiments were Anti‐GFP polyclonal antibody (Proteintech, 50430‐2‐AP), actin (plant specific) mouse monoclonal antibody (ABclonal, AC009), goat antimouse IgG‐HRP conjugated (Easybio, BE0102), goat antirabbit IgG‐HRP conjugated (Solarbio, SA134). siRNA was synthesized by Sangon Biotech (Shanghai) Co., Ltd. See Tables S1 and S2 (Supporting Information) for all RNA sequences and primers used in this study.

### General Methods

The size and morphology of nanoparticles were investigated by transmission electron microscopy (TEM, JEM‐1400) with an operating voltage of 120 kV. The dynamic light scattering (DLS) size was examined by Zetasizer pro‐blue (Malvern) at room temperature. To prepare the 16 C line *N. benthamiana* leaves for confocal imaging, a small leaf section was cut and mounted between glass slides, and then the samples were observed on laser confocal scanning microscopes (Zeiss LSM980 with Airyscan 2). Images were obtained at 20× magnification. The same imaging parameters and quantification analyses were performed on all samples.

### Preparation of MSNs

CTAB (0.29 g) was dissolved in 150 mL ddH_2_O and NH_3_.H_2_O (1.39 mL) was added to the solution. This aqueous solution was heated to 50 °C and kept stable for 60 min, followed by adding 2.6 mL mixture (100 µL APTS, 491 µL TEOS, 2009 µL ethanol) drop‐wise into the solution while stirring vigorously for 1 h. Then the reaction was stopped and aged at 50 °C for 20 h. For acquiring larger‐sized MSNs, the amount of NH_3_.H_2_O was increased to 2.78, 4.17, and 5.56 mL, respectively. Once the solution was cooled, the as‐synthesized nanoparticles were centrifuged and washed with ethanol. The CTAB was removed by using a hydrogen chloride and ethanol mixture.

### MSN‐siRNA Binding and Nuclease Protection Assay

To coat MSNs with siRNA for delivery, 1 µg of siRNA was mixed with MSNs at various mass ratios in delivery buffer (25 mm MES, 15 mm MgCl_2_ at pH 6.0). The mixture was immediately mixed upside down for 5–10 s, and then incubated for 60 min at 4 °C. Some of the complex solution was loaded onto a 2% agarose gel (gel red was added to the agarose gel in advance), with free siRNA as a reference. After gel electrophoresis (80 V for 20 min), RNA bands were visualized by the Chemidoc imaging system (Biorad).

For the nuclease protection assay, free siRNA (600 ng) and 600 ng siRNA on MSNs were separately incubated with 1 µL RNase A at 37 °C for 1 h. After incubation, all siRNA were desorbed from MSNs at 95 °C for 1 h in the presence of 1.6% SDS and 0.8 m NaCl. Desorbed siRNA and treated free siRNA were run on a 2% agarose gel with 600 ng siRNA as a reference.

### MSN Degradation Test

MSNs were suspended in *N. benthamiana* leaf cell lysate at a concentration of 1 mg mL^−1^ and placed at room temperature. Then particles were settled by centrifugation and suspended in water at the designated time point. The silica solution was analyzed by TEM imaging to visualize the morphology of MSNs during degradation.

### Plant Growth

The plants used in this study included the wild type of *N. benthamiana* and transgenic 16C GFP *N. benthamiana* (Voinnet and Baulcombe, 1997). These *N. benthamiana* seeds were kindly provided by the Caixia Gao Lab, Institute of Genetics and Developmental Biology, Chinese Academy of Sciences. The seeds were germinated on Murashige and Skoog (MS) medium for a week after being sterilized by 70% ethanol and 2–3% NaClO, and transferred to a soil mixture for 4 weeks before experiments. *N. benthamiana* plants were maintained at 25 °C with the daylight of 12 h at 100 µmol photons m^−2^ s^−1^ and a 12 h dark cycle.

### TEM for Tracking of MSNs in *N. benthamiana* Leaves

To study MSN internalization leaves infiltrated or sprayed with MSN‐siRNA complex were cut into small pieces ≈3 mm × 3 mm in size. The leaf samples were fixed using 2% glutaraldehyde overnight and then subjected to self‐made vacuum equipment to remove air in the vacuoles. The samples were rinsed with PBS and postfixed with 1% osmium for 2 h. After rinsing with PBS, the samples were processed to gradient dehydration with different concentrations of ethanol and then with acetone. Finally, the samples were transferred into epoxy resin for embedding and solidified at 37, 45, and 70 °C. The epoxy resin‐embedded samples were sectioned using a LEICA EM UC7 ultramicrotome (Leica UC7) and the sections were stained with 2% uranyl acetate for 15 min, following imaging by TEM at an acceleration voltage of 120 kV.

### 4.1 *N. benthamiana* Leaves Treated with MSN‐siRNA via Infiltration and Spray

Healthy seedlings (4–6 weeks old) of *N. benthamiana* and the 16 C line were selected for the study. For infiltration treatment, a small puncture on the abaxial (bottom) surface of the leaf was introduced with a pipette tip, and ≈100 µL of the MSN‐siRNA solution with 0.03% tween 20 was infiltrated through the puncture area gently by using a 1 mL needleless syringe. According to the previous study,^[^ ^12^ ^]^ to keep the siRNA from degradation, the MSN:siRNA ratio by finding the minimum amount of MSN was optimized to completely load the given amount of siRNA. The MSN‐siRNA solution was prepared as follows: 10 µg siRNA and 300 µg MSN were mixed in 1 mL delivery buffer for 1 h, and the final concentration of siRNAs in the formulations was 10 µg mL^−1^. Treated leaves were collected for total RNA extraction at 1 day post‐treatment in the study of *GFP* and *ROQ1* gene knockdown validation test. The control groups were delivery buffer with MSN and scrambled siRNA, and delivery buffer with free siRNA of the corresponding gene. In the spraying process, the MSN‐siRNA solution was also prepared as follows: 10 µg siRNA and 300 µg MSN were mixed in 1 mL delivery buffer for 1 h, then 1 mL solution of MSN‐siRNA with 0.3% tween 20 was loaded into a 2 mL spray atomizer, the spray nozzle was set 2 cm away from the leaf, and the solution was sprayed until the spray mist covered the entire leaf area. The subsequent qPCR analysis procedure was consistent with the infiltration process.

In the further silencing study of *GFP*, *PDS*, *ChlH*, *SOS*, *HHL1*, and *FtsH2* genes, *N. benthamiana* leaves were sprayed with relevant MSNs‐siRNA, and the collection time of leaves extended to 1, 3, and 5 days post‐treatment, followed by mRNA transcription level analysis. For sequential spraying treatments, leaves were sprayed every other day with MSN‐siRNA solution with 0.3% tween 20 from day 0 to day 13 for 2 weeks, and leaves were harvested at day 4, 7, 11, and day 13 postspraying.

### RNA Extraction, RT‐PCR, and Quantitative PCR

Total RNA was extracted from *N. benthamiana* leaves using Trizol reagent (Vazyme) according to the manufacturer's protocol. The RNA samples were reverse transcript to first strand cDNA using a PrimeScript RT reagent kit (Vazyme). For qPCR, GAPDH was used as an internal reference gene. Quantitative real‐time RT‐PCR was performed using gene‐specific primers and SYBR Premix ExTaq reagent (Vazyme) with a real‐time RT‐PCR system (Applied Biosystems 7500/7500 Fast Real‐Time PCR Systems) following the manufacturer's instructions. *N* = 3 replicates are three independent repeated experiments, and expression levels were normalized against the GAPDH gene.

### Western Blot

*N. benthamiana* 16 C line which stably expresses GFP was used for direct visualization of protein expression changes by applied MSN‐siGFP. The total protein was isolated on ice by plant protein extracts (Solarbio) when the leaves were harvested at 1, 3, and 5 days post‐treatment. The extracted protein was quantified by a BCA protein concentration determination kit (Beyotime). Afterward, a western blot was carried out with the primary anti‐GFP antibody (dilution 1:2000, Proteintech) as required. Anti‐plant actin mouse monoclonal antibody was used to detect reference protein (dilution 1:2000, Abclonal). Densitometric quantification was performed using Image J64 software. The representative result is presented from four independent experiments.

### *N*. *benthamiana* Leaf Phenotypic Change

*HHL1* and *FtsH2* were photo‐damage repair relative genes. In the phenotypic validation experimental section, all experimental seedlings treated with MSN‐siRNA complex that targets the *HHL1* or *FtsH2* gene were grown under high light conditions of 1300 µmol photons m^−2^ s^−1^, 16 h light, and 8 h dark at 23 °C cycle. And the changes in the *N. benthamiana* leaf were photographed till 14 days post‐treatment.

### Validation of Multiple Gene Silencing by Digital PCR

For validation of the silencing efficiency of multiple genes by using MSNs‐siRNAs. The siRNA that targets *HHL1*, *FtsH2*, *SOS*, and *GFP* was incubated with MSN independently. Then the solutions were mixed and sprayed on 16C GFP *N. benthamiana* leaves every 2 days for 6 days. The seedlings were grown under high light conditions of 1300 µmol photons m^−2^ s^−1^, 16 h light, and 8 h dark at 23 °C cycle, the *N. benthamiana* leaves were photographed to catch changes. The cDNA reverse transcript from the total RNA of multiple MSNs‐siRNAs‐treated *N. benthamiana* leaves was used for digital PCR analysis. The mRNA of the target gene expression was detected by their short primers and corresponding Taqman probes. All four target genes could be detected in one chip. The sequence and probe marker are listed in Table S3 (Supporting Information). qPCR was also carried out for mRNA transcription level analysis.

### Statistical Analysis

Data were analyzed with GraphPad Prism Version 6.01, Image J‐win64. For qPCR analysis, *N* = 3 replicates were three independent experiments. Each qPCR in three independent experiments was performed in triplicate. mRNA level change data were expressed as each mean from the three independent experiments, together with error bars indicating SEM. Significance was measured with one‐way ANOVA tests. Probabilities of *p*  < 0.05 were considered significant with asterisks in figures denoting *P* values as follows: ^*^ *p* < 0.05, ^**^ *p* < 0.01, ^***^ *p* < 0.001, ^****^ *p* <  0.0001.

## Conflict of Interest

The authors declare no conflict of interest.

## Supporting information

Supporting Information

## Data Availability

The data that support the findings of this study are available from the corresponding author upon reasonable request.
