# Supplementary material for: Mesoporous Silica Nanoparticles Mediate SiRNA Delivery for Long‐Term Multi‐Gene Silencing in Intact Plants
Source: Adv Sci (Weinh). 2023 Dec 25;11(9):2301358. doi: 10.1002/advs.202301358 (PMC10916655; doi:10.1002/advs.202301358)
Supplement: Supplementary file 1 — Supporting Information [file ADVS-11-2301358-s001.pdf]

ADVANCED SCIENCE
Open Access

Supporting Information

for *Adv. Sci.*, DOI 10.1002/advs.202301358

Mesoporous Silica Nanoparticles Mediate SiRNA Delivery for Long-Term Multi-Gene Silencing in Intact Plants

Yao Cai, Zhujiang Liu, Hang Wang, Huan Meng and Yuhong Cao**

Supporting Information

Mesoporous silica nanoparticles mediate siRNA delivery for long-term multi-gene silencing in intact plants

Yao Cai, Zhujiang Liu, Hang Wang, Huan Meng, Yuhong Cao**

Dr. Y. Cai
Key Laboratory for Biomedical Effects of Nanomaterials and Nanosafety, National Center for Nanoscience and Technology, Chinese Academy of Sciences, Beijing 100190, China.

Dr. Z. Liu
Key Laboratory for Biomedical Effects of Nanomaterials and Nanosafety, National Center for Nanoscience and Technology, Chinese Academy of Sciences, Beijing 100190, China.
Email: liuzj2021@nanoctr.cn

Dr. H. Wang
Key Laboratory for Biomedical Effects of Nanomaterials and Nanosafety, National Center for Nanoscience and Technology, Chinese Academy of Sciences, Beijing 100190, China.

Prof. H. Meng
Key Laboratory for Biomedical Effects of Nanomaterials and Nanosafety, National Center for Nanoscience and Technology, Chinese Academy of Sciences, Beijing 100190, China.

Prof. Y. Cao
Key Laboratory for Biomedical Effects of Nanomaterials and Nanosafety, National Center for Nanoscience and Technology, Chinese Academy of Sciences, Beijing 100190, China; University of Chinese Academy of Sciences, School of NanoScience and Technology, Beijing 100049, China.
Email: caoyh@nanoctr.cn

Keywords: mesoporous silica nanoparticles, siRNA delivery, gene silencing, plant biotechnology

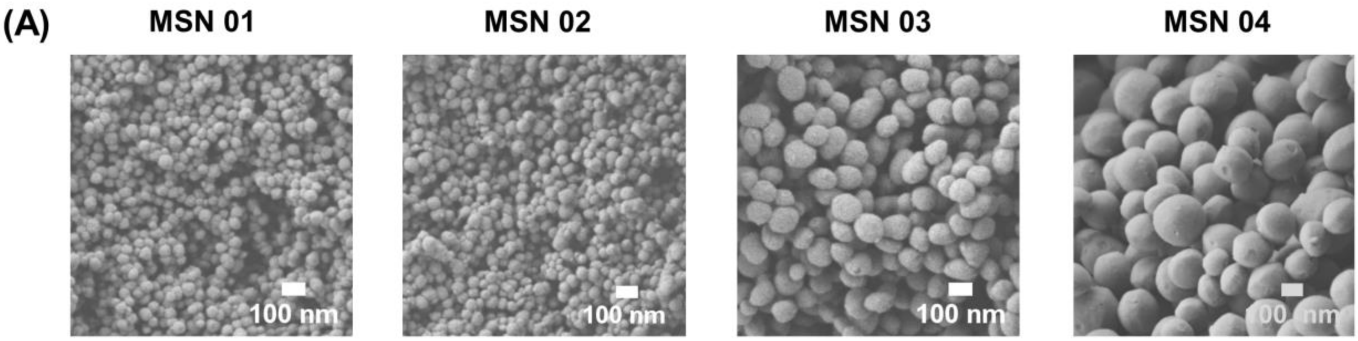

(B)

samples	diameter DLS (nm)	PDI	diameter TEM (nm)
MSN 01	44.9	0.03	31±6.4
MSN 02	60.7	0.14	53±6.2
MSN 03	152.5	0.15	99±4.5
MSN 04	301.2	0.03	193±9.6

Figure S1. Characterization of MSNs. (A) Representative SEM images of four synthesized MSNs with different sizes. (B) Diameter determination of four different sized synthesized MSNs.

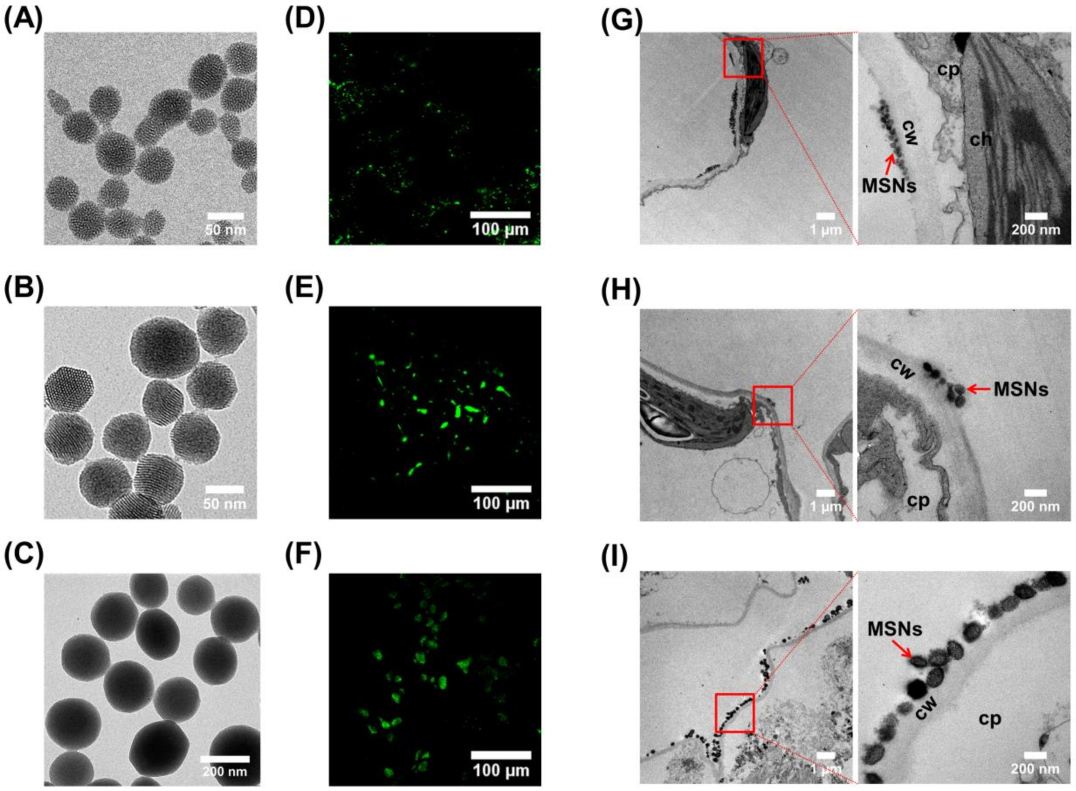

Figure S2. Analysis of MSNs internalization in *N. benthamiana* leaves. Representative TEM images of (A) MSN 02 (53 nm); (B) MSN 03 (99 nm); (C) MSN 04 (193 nm). Scale bars from A

to C, 50 nm, 50 nm and 200 nm. Representative confocal microscopy images of *N. benthamiana* leaves infiltrated with three different sized MSNs loaded with FAM-modified scramble siRNA, the fluorescence was collected to analyze the distribution of MSNs in leaves. (D) MSN 02; (E) MSN 03; (F) MSN 04. Scale bars: 100 μm. Representative TEM images of *N. benthamiana* leaves 24 h post-infiltration with siRNA-functionalized MSNs with diameters of (G) MSN 02; (H) MSN 03; (I) MSN 04. Scale bars: 1 μm. The images show magnifications on the right, with the red boxes indicating the areas of magnification. Scale bars: 200 nm. The red arrows indicate MSNs. Annotations represent cell wall (cw), cytoplasm (cp) and chloroplast (ch).

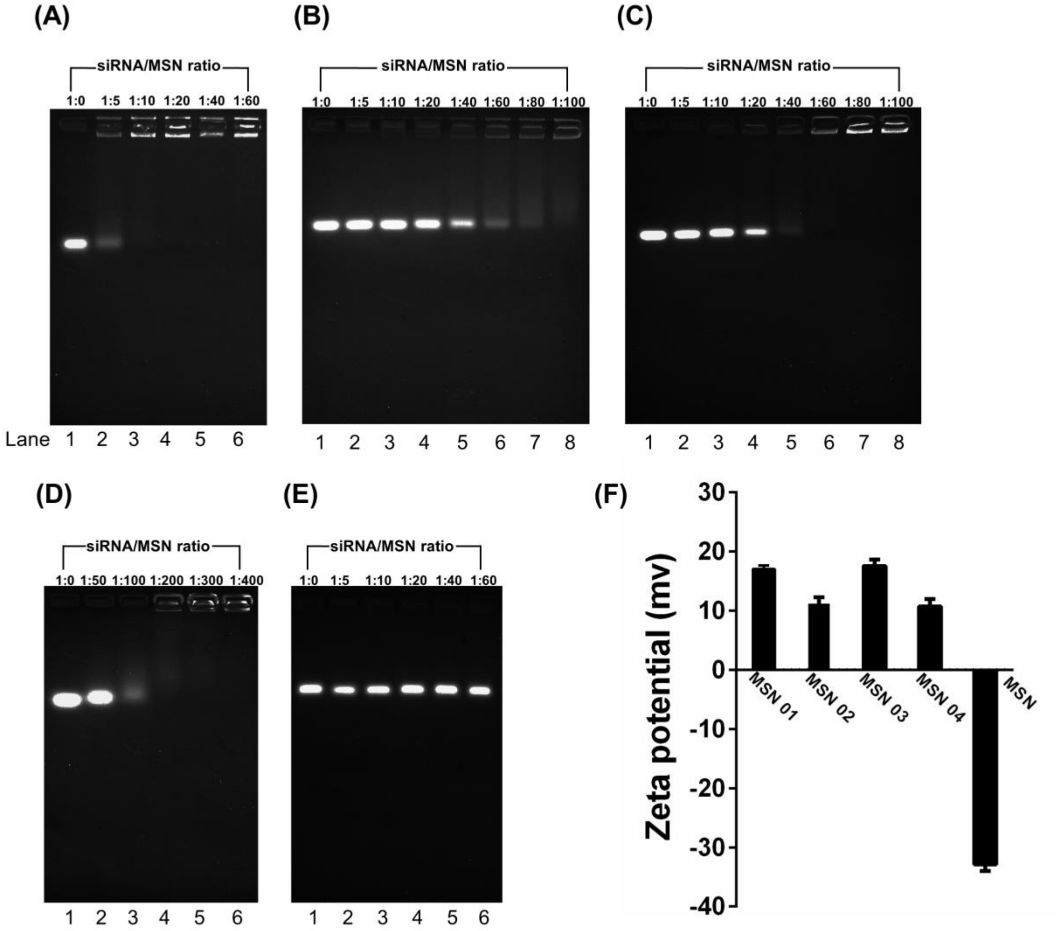

Figure S3. Different sized MSNs loading capacity estimation. (A) MSN of 31 nm incubated with siRNA in different ratios for 1 h at 4℃, the siRNA/MSN with ratio of 1:10 was at saturation. (B) MSN of 53 nm diameter incubated with siRNA in different ratios for 1 h at 4℃, the siRNA/MSN ratio of 1:80 was at saturation. (C) MSN of 99 nm diameter incubated with siRNA in different ratio for 1 h at 4℃, the siRNA/MSN ratio of 1:40 was at saturation. (D) MSN of 193 nm diameter incubated with siRNA in different ratios for 1 h at 4℃, the siRNA/MSN ratio of 1:200 was at saturation. (E) MSN of 31 nm diameter without APTS modification incubated with siRNA, siRNA could not be combined. (F) Zeta potential of MSN 01, MSN 02, MSN 03, MSN 04 and bare MSN.

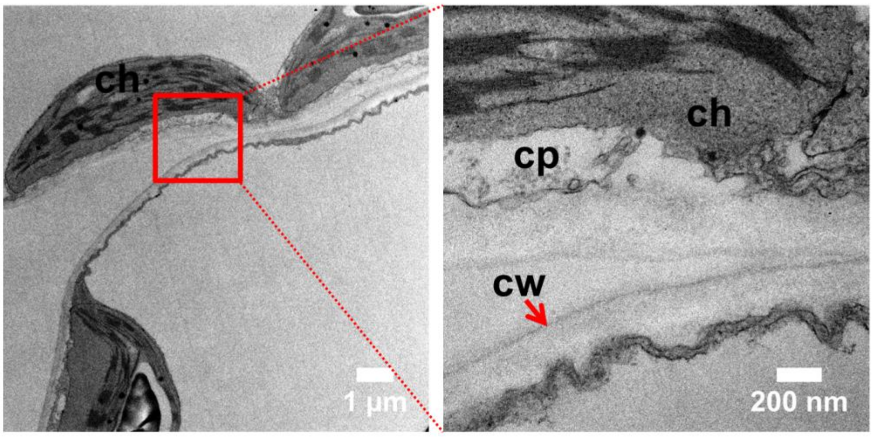

Figure S4. TEM images of normal *N. benthamiana* leaf tissue. Representative TEM images of *N. benthamiana* leaves infiltrated with delivery buffer. Annotations represent cell wall (cw), cytoplasm (cp) and chloroplast (ch).

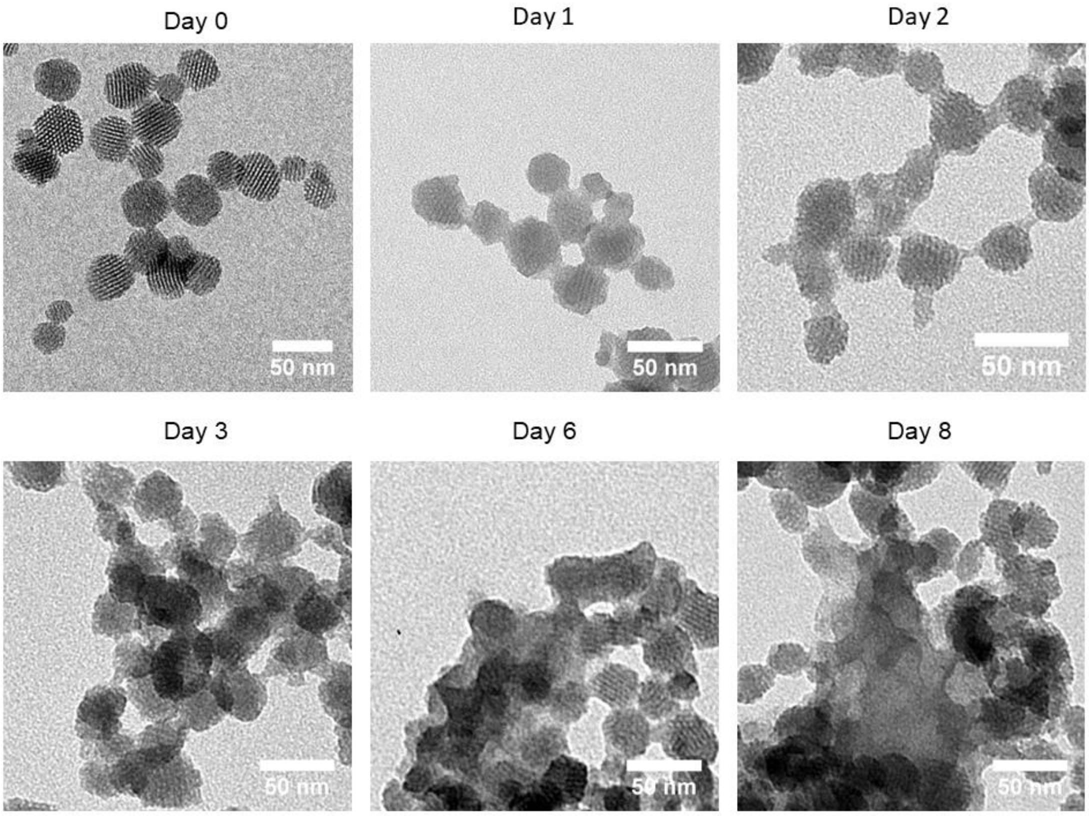

Figure S5. MSN degradation by plant cell lysate. Representative TEM images of MSN incubated with plant cell lysate from day 0 to day 8. All scale bars, 50 nm.

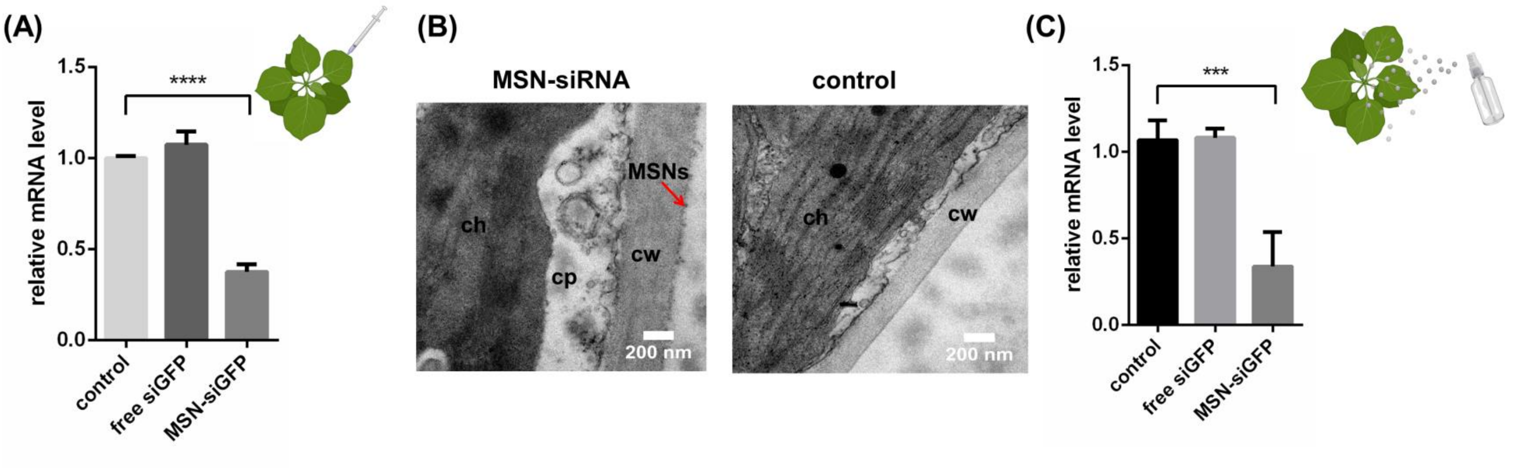

Figure S6. ***N. benthamiana*** **leaves infiltrated or sprayed with MSN-siGFP.** (A) qPCR analysis of infiltrated MSN-siGFP effects on *GFP* expression. (B) Foliar application of siRNA into *N. benthamiana* leaf cells mediated by MSNs. The MSN-siRNA complexes were applied to leaves using a spray atomizer, and representative TEM images of control *N. benthamiana* leaves sprayed with buffer without MSN and *N. benthamiana* leaves 24 h after spraying with siRNA-functionalized MSNs. Annotations represent cell wall (cw), cytoplasm (cp) and chloroplast (ch). (C) qPCR analysis of sprayed MSN-siGFP effects on *GFP* expression. ***P < 0.001 and ****P < 0.0001 are significant using one-way ANOVA test. Data represent mean ± sem.

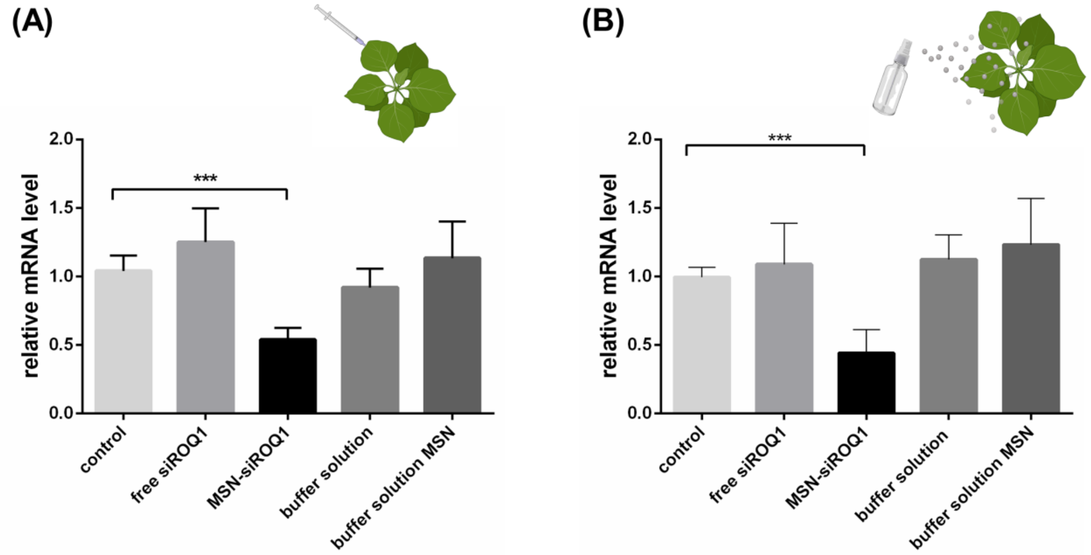

Figure S7. ***N. benthamiana*** **leaves infiltrated or sprayed with MSN-siROQ1.** (A) qPCR analysis of infiltrated MSN-siROQ1 effects on *ROQ1* expression. (B) qPCR analysis of sprayed MSN-siROQ1 effects on *ROQ1* expression. ***P < 0.001 is significant using one-way ANOVA test. Data represent mean ± sem.

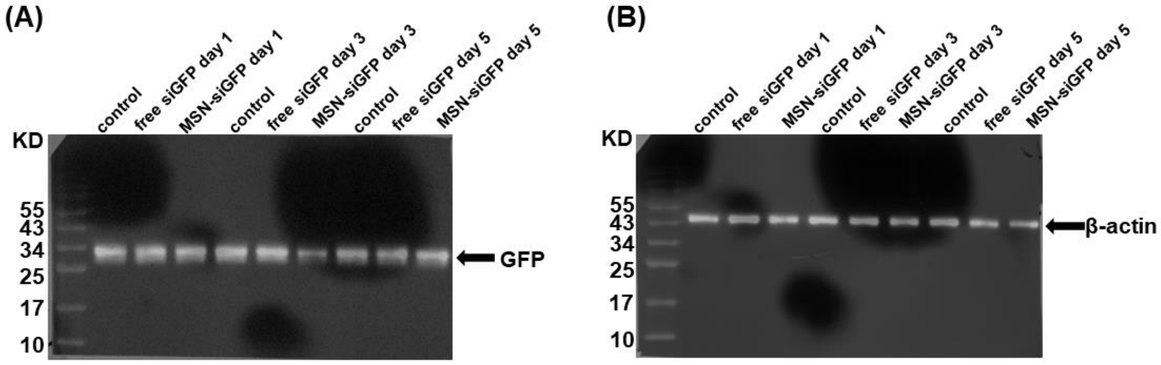

Figure S8. GFP western blot results by spray treatment. (A) Western blot for GFP extracted from control, free siGFP and MSN-siGFP delivered 16C line leaves at 1, 3 and 5 days post spraying. (B) Western blot of the plant β-actin protein.

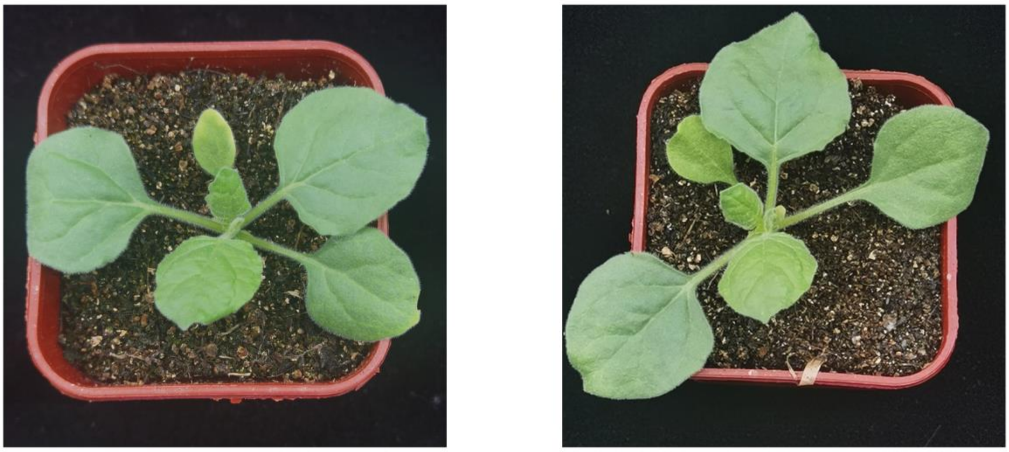

Figure S9. *N. benthamiana* phenotype changes post spraying of MSN-siHHL1 (left) and MSN-siFtsH2 (right) without high light exposure.

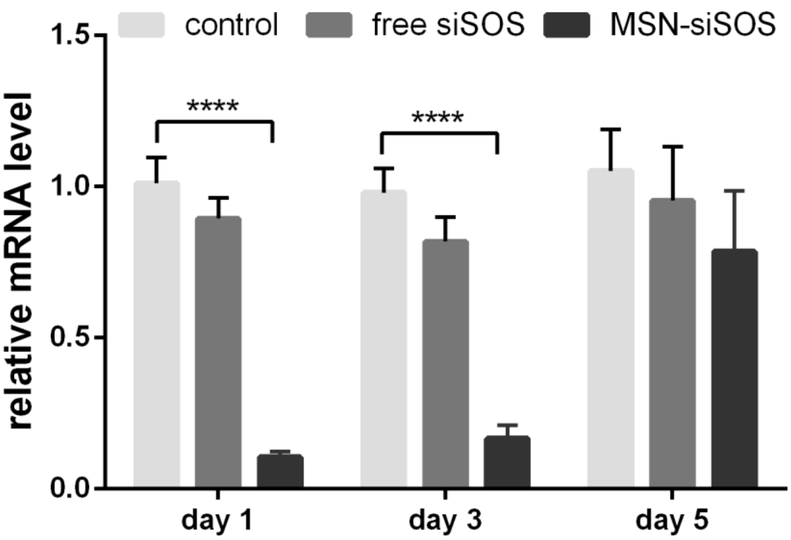

Figure S10. qPCR analysis for SOS mRNA fold changes at 1, 3 and 5 days post-treatment by spraying MSN-siSOS. ****$P < 0.0001$ is significant using one-way ANOVA test. Data represent mean ± sem.

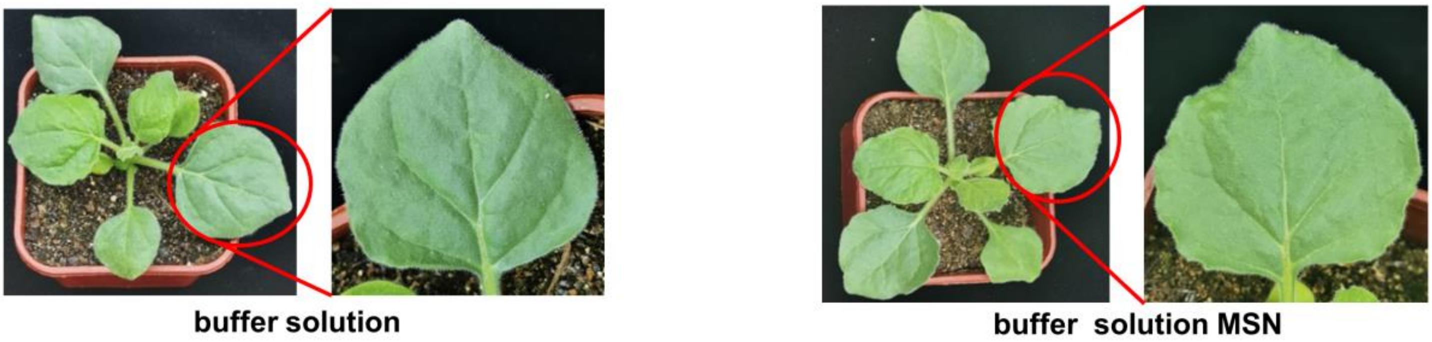

Figure S11. ***N. benthamiana*** **phenotype changes post spraying of buffer solution and buffer solution with MSN.**

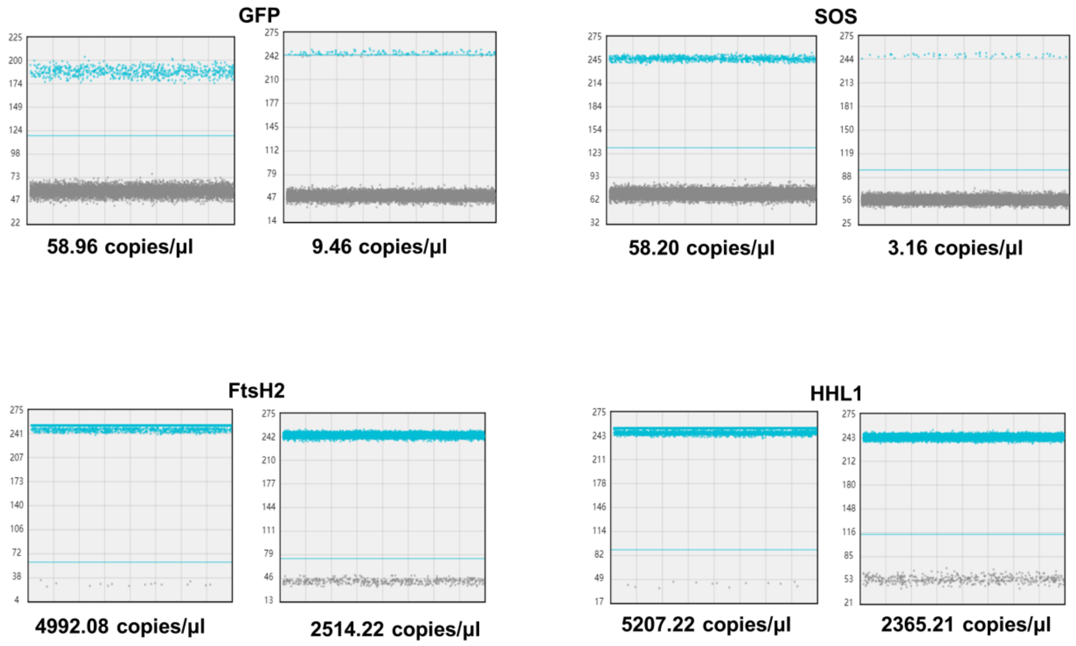

Figure S12. Digital PCR analysis of fold changes for four mRNAs (FtsH2, HHL1, SOS, GFP) after spraying the multiple MSN-siRNAs.

Table S1. siRNA sequences list

gene name	sense (5'-3') sequence	antisense (5'-3') sequence
ROQ1	GGUUUAAUUUGGUGUAUA A	UUAUACACCAAAUUAAACC
GFP	GGCAUCAAAGCCAACUUCAAAA	UUGAAGUUGGCUUUGAUGCCGU
PDS	UACUGAAGCAGUCACCAAGAAUCTA	UAGAUUCUUGGUGACUGCUUCAGUAAG
ChlH	GGAAGCUCAGUACCAAUCAUCACTT	AAGUGAUGAUUGGUACUGAGCUUCCAA
FtsH2	CAACCGUGAAGCAAUUGAUAAGATT	AAUCUUAUCAAUUGCUUCACGGUUGUU
HHL1	CUGCAGAACUUAGAUGUCACAUGGA	UCCAUGUGACAUCUAAGUUCUGCAGUA
SOS	GCUUGAAGCAGGAACCUGAAUCAGA	UCUGAUUCAGGUUCCUGCUUCAAGCUC
Scrambled siRNA	UUCUCCGAACGUGUCACGUTT	ACGUGACACGUUCGGAGAATT

Table S2. RT-PCR primer sequences list

name	primer
GAPDH-F	AGCTCAAGGGAATTCTCGATG
GAPDH-R	AACCTTAACCATGTCATCTCCC
ROQ1-F	TCC CCG ACA TAA AGG AAT GC
ROQ1-R	GTC CCC TGG ACT CAA ACA GG

GFP-F	GACCACATGAAGCAGCACGA
GFP-R	CGGGTCTTGTAGTTGCCGTC
PDS-F	GATGCAGTGCATTTTGATTGCTTT
PDS-R	CATTTATCACAGGAACTCCCACTAG
ChlH-F	GCTAATGCTCAGGTACGAACG
ChlH-R	CTCAATCTCACGAACTCCCTC
FtsH2-F	GGACTTGGAGGAAGAGCTGC
FtsH2-R	TAGCCAAACCGGTGATCTGC
HHL1-F	GTACCAACGTATGGCTCCTCCTA
HHL1-R	TGGCCATGCGAACAAAGATA
SOS-F	AGGAGCAGAAGCCTCGTATCA
SOS-R	GTCTGTGGTCGCAGCAGAAG

Table S3. Taqman probes sequences

taqman probe name	sequence	5' modification	3' modification
SOS	TCAGCGTTCCCCAGACAAACCGGAGGT	CY5	MGB
FtsH2	ACCACTGGTGCTGCAGGCGATTTGC	HEX	MGB
HHL2	CCAAGATTGAAGATGATGGCAACCCCA	ROX	MGB
GFP	TTCAAGTCCGCCATGCCCGAAGGCT	FAM	BHQ2
